# A Self-Controlled and Self-Healing Model of Bacterial Cells

**DOI:** 10.3390/membranes12070678

**Published:** 2022-06-30

**Authors:** Max Garzon, Petr Sosik, Jan Drastík, Omar Skalli

**Affiliations:** 1 Department of Computer Science, The University of Memphis, Memphis, TN 38152, USA; mgarzon@memphis.edu; 2 Research Institute of the IT4Innovations Centre of Excellence, Silesian University in Opava, 74601 Opava, Czech Republic; jan.drastik@fpf.slu.cz; 3 Department of Biology, The University of Memphis, Memphis, TN 38152, USA; oskalli@memphis.edu

**Keywords:** membrane self-assembly, protein transport channels, prokaryotic cell division, morphogenetic system, bacterial kinetics, self-controlled growth, self-healing

## Abstract

A new kind of self-assembly model, morphogenetic (M) systems, assembles spatial units into larger structures through local interactions of simpler components and enables discovery of new principles for cellular membrane assembly, development, and its interface function. The model is based on interactions among three kinds of constitutive objects such as tiles and protein-like elements in discrete time and continuous 3D space. It was motivated by achieving a balance between three conflicting goals: biological, physical-chemical, and computational realism. A recent example is a unified model of morphogenesis of a single biological cell, its membrane and cytoskeleton formation, and finally, its self-reproduction. Here, a family of dynamic M systems (M_bac_) is described with similar characteristics, modeling the process of bacterial cell formation and division that exhibits bacterial behaviors of living cells at the macro-level (including cell growth that is self-controlled and sensitive to the presence/absence of nutrients transported through membranes), as well as self-healing properties. Remarkably, it consists of only 20 or so developmental rules. Furthermore, since the model exhibits membrane formation and septic mitosis, it affords more rigorous definitions of concepts such as *injury* and *self-healing* that enable *quantitative* analyses of these kinds of properties. M_bac_ shows that self-assembly and interactions of living organisms with their environments and membrane interfaces are critical for self-healing, and that these properties can be defined and quantified more rigorously and precisely, despite their complexity.

## 1. Introduction

The emergence of the macro-world from interactions at the micro-world is a fundamental problem in modern biology. Self-assembly is a solution and has become a subject of its own. A fundamental property of living organisms with complex shapes and properties is that they are assembled from simpler inert materials and subunits through complex morphogenetic processes. For instance, most virus species assemble one or more protein types around a nucleic acid molecule, and this assembly is governed by the nature of their mutual interactions. This kind of assembly is arguably the simplest biological morphogenetic process. Several levels above in complexity are bacteria. These organisms are composite assemblies with multiple polymeric structures, each of which arises from molecular subunits, again through specific morphogenetic processes. For instance, in its simplest form, the bacterial envelope is a combination of lipid subunits assembling into a membrane and glycoprotein subunits assembling into the outer envelope. Eventually, even more complex morphogenetic processes lead to the assembly of multicellular organisms. For these organisms, the cell is the fundamental unit. Division processes, such as mitosis, generate more units (cells) which integrate with each other through specific interactions to eventually generate a *dynamic* organism with specific shape and properties.

These considerations point to what is perhaps the most fundamental and difficult question in biology: *how can nonliving parts come together into an organized complex structure that could be considered alive?* The goal of this paper is to address some aspects of this question from a mathematical and computational standpoint by asking, more specifically, whether it is possible to characterize the contribution that self-organization, such as computational self-assembly and morphogenesis, makes to help answer this question. Special emphasis is placed on the role of the membrane, its topology, and its transport and interface functions controlling the assembly process.

Alan Turing, the founder of modern computer science, addressed a particular case of this question very early in his pioneering work [1] before specific explanations of heredity and information processing were revealed by the discovery of the structure of DNA [2], by asking about the nature of the process that produces and maintains the spot pattern on a leopard’s skin. Building computational models of morphogenesis has been explored ever since in a variety of ways and directions. They range from highly specific and arguably physically realistic models for specific morphogenetic mechanisms or organisms [3,4,5,6], to general approaches and models of entire biological units such as a living cell or a full organism [7,8], to broadly encompassing theoretical frameworks to address this fundamental question [9,10].

According to a central tenet in biology, the living cell is the fundamental biological unit. Cells can exist independently of others, as in bacteria or ciliates, or they can assemble into organisms. New cells are produced from existing ones through cell division, a process consisting of successive steps specific to the cell type (e.g., prokaryotes or eukaryotes, somatic or germ cells in [11]). One of the simplest processes of cell division is the binary fission of bacteria [12]. Binary fission begins with the replication of the DNA located inside the cell and is followed by the segregation of the two DNAs towards opposite ends of the cell. Subsequently, two opposite sides of the cell wall pinch inward toward each other and form a septum when they meet. This septum divides the bacteria into two compartments of roughly equal volume, each containing a copy of the DNA. The septum then splits down the cell through the middle to yield two daughter cells and complete the reproductive cycle.

Much is known about the specific molecular events leading to bacterial cell division [13,14]. Computational modeling based on empirical data about these events has moved the field toward a comprehensive understanding of the regulatory networks at play during the bacterial cell cycle [15]. Although a bacterium is the simplest biological system with a metabolism, they are already highly complex assemblies consisting of a myriad of molecular components. Bacterial fission also poses a challenging problem for synthetic biology [16]. Thus, there is still a long road ahead to produce a synthetic system that would emulate bacterial fission realistically.

Motivated by these considerations, [17,18] recently proposed a dynamic computational model, M systems, based in part on the techniques of membrane computing [19]. M systems arose as an evolution of so-called membrane systems (or P systems, [19]), which generally consist of a structure of (possibly nested) delimiting *regions* in 2D or 3D space enclosed by membranes, as illustrated below, and as a mathematical abstraction of common biological structures. In addition, the system contains objects from a certain set *O*, placed inside membranes or in their surrounding environment, which abstractly represent physical elementary objects such as molecules or antibiotics or nutrients, but which do not bear any information about their location or shape within the membranes. Multiple copies of the same object can be present in a region, so they work with *multisets* of objects because multiplicity carries weight biologically. P-systems may also include other objects inspired by biological proteins, which are primary carriers of biological function by self-folding into characteristic shapes. Protein abstractions have been explicitly used in P systems [20,21]. They interact through a number of *production rules* in the chemical/computational sense, i.e., they operate on certain reagents/objects within a membrane to produce other objects, perhaps in an adjacent membrane. These rules can be thus construed as *abstractions of ordinary chemical reactions* among elementary objects that catalyze the production of other products necessary for a cell to function biologically.

These systems capture the principles of biological morphogenetic processes, yet can be understood in terms of a few abstract but simple local rules of interaction that cause them to unfold into membrane structures exhibiting complex emerging properties, as explained in Section 2. Later, [22] presented a simulation framework for M systems called *Cytos*, and used it to build an M system that emulates bacterial cell formation, growth, and division by fission. The goal of this work is to introduce and further explore emerging properties of an M system, generalizing it for the formation of more complex biological assemblies, such as bacterial cells. We describe a family of M systems of a bacterial cell population that exhibit growth kinetics as emergent properties that follow very closely their biologically counterparts. Section 3 shows M system models of three representative species of bacteria. Section 4 discusses an abstract and principled definition of self-healing and robustness properties of M. Finally, Section 5 discuss the significance of our results and outlines questions of interest for further research.

## 2. Materials and Methods

This Section describes M systems in detail and the biological data used to assess the models.

M systems combine and extend P-systems and self-assembly systems [23] along three different directions. First, they *do not presuppose any specific membrane structure* or even membranes (as is the case in P-systems), but rather provide certain production rules that will enable their assembly, including the structure and layout of the membranes themselves. Second, they explicitly assume that objects in the system possess geometric features as well, such as shape and position, in addition to their self-assembly capabilities, based on simpler component objects. This is in recognition of the important role that shape and physical location play in ordinary biology, usually not explicitly factored in most kinds of biological models. To avoid misunderstanding by biologists and biochemists, a new term “protion’’ has been introduced to denote such abstract objects in M systems in (Sosik et al., 2018). Third, the primary goal in studying M systems, as given by sets of elementary objects with their rules of interaction in a host geometric space (e.g., ordinary 2D or 3D space), is to examine and characterize the behavior of larger and more complex objects and systems *emerging* as a result of the interaction of component objects according to a given set of reaction/production rules and environmental conditions they are placed in, including nutrients.

### 2.1. Computational Methdology and Simulation

A 3D *M system* is embedded in a 3D Euclidean (ordinary) space and is defined by specifying three types of parameters as objects present in the system:

- *Floating objects* are small shapeless atomic objects of positive volume floating freely within the environment and occupying a specific position in space at every moment (akin to a center of gravity or centroid). They can be carried through tiles via protion channels and participate in mutual reactions with other types of objects, as specified by the production rules.
- *Tiles* are obstructive (like solid) objects of dimension 1 or 2. Each tile has its own pre-defined shape and size. Unlike other models of self-assembly systems, such as the aTAM model [23,24], tiles are not present implicitly in arbitrarily many copies, but can only be created via reactions involving floating objects. Selected points or segments on tiles called *connectors* are covered with certain types of *glues*. Another tile can stick to a connector by its own connector with a matching glue, where a *match* is defined by a glue relation pre-specified in the model. In this way, larger interconnected structures of tiles can gradually self-assemble into more complex objects.
- *Protions* are regulators or catalysts that (i) allow for certain reactions and (ii) transport floating objects through a tile (which may be part of a wall of a self-assembled closed compartment, akin to an ion channel in a biological membrane). They have a certain unchangeable position on a tile. Protions follow the notion in P systems as abstractions of proteins on membranes [20]. The connected tiles can be also disconnected and/or destroyed by rules under certain conditions requiring the presence of specific floating objects.
- *Reaction rules* can only be of four types: *metabolic*, *creation*, *destruction*, and *division* rules, as defined by the kind of result they may produce when applied to objects in the system.
- *Brownian motion* moves objects by a certain distance (as specified by a parameter in the system, the interaction radius) at every iteration, before applying any rules. It ensures that further interaction is eventually possible, even if no rule was applied at a given time.

A full formal definition of an M system can be found, e.g., in [22], available online at http://sosik.zam.slu.cz/msystem/CMC20.pdf (accessed on 20 May 2022). The most important component of an M system is the set of reaction rules *R* that specify (in abstract) the interaction between the elementary objects in the model. They play the role of *physical or chemical laws* that determine interactions between objects. A typical reaction rule has the form *u* → *v*, where *u* (reactants) and *v* (products) are multisets which may contain floating objects, protions, and/or tiles. A more detailed, but also more technical, description of M systems can be found in [17,18].

A nontrivial example of such an M system is described in [17]. This system produces a model M_0_ of a living cell self-assembled from basic pentagonal tiles and undergoing a sort of morphogenetic process to generate a cytoskeleton in its interior, eventually producing a copy of itself, akin to a mitotic division. *The salient properties of M_0_ are that the specific shape of the self-assembled cell, its cytoskeleton, and the ability to self-replicate are not explicitly defined by the rules*, *nor is the ability to form a cytoskeleton*, *let alone self-replicate*. These structures and properties are *emergent properties* of the system that are, in principle, hard to surmise just from the original basic objects, protions, and rules of interaction specified in the system, as can be seen in an animation [25]. This and other examples will be described in a bit more detail in the next Section. The M system M_0_ is illustrated below and its technical description can be found in detail online at the M systems web page, http://sosik.zam.slu.cz/msystem/Cytos-PS10-Appendix.pdf (accessed on 20 May 2022).

### 2.2. Comparison to Other Approaches to Cell Growth Modeling

Classical quantitative mathematical models of cell population dynamics were based on Ordinary Differential Equations (ODEs), as in [26], without explicit relations to spatial constrains, and therefore, they were limited to rather simple cases of matching qualitatively the phenomenon of interest (e.g., exponential growth profile), coarsely at best. Later, the importance of spatial and temporal constraints and control for the growth and formation of complex cell colonies has been recognized and models based on cellular automata-like approaches emerged [27,28]. Individual-based modeling (IbM) became an important tool for quantitative modeling in biology, based on actions of individual agents and interactions among them and with their environment, including explicit spatio-temporal dynamics [29,30].

In contrast with most IbM applications in microbiology focused on population dynamics, treating individual cells as atomic units with certain parameters, the M systems model starts with a single cell model and generates other objects in the system through dynamic interactions between individual components. Macro-phenomena such as cell division and mitoses *emerge as a result of their interactions*, rather than being posited to occur at hypothetical times. The result is a dynamic of populations of objects mostly self-controlled and influenced by external factors such as nutrient concentration and/or antibiotics. Computational models of a single cell of *E. coli* growth were described in, e.g., [31,32]. These models are based, however, on complex qualitative descriptions of cells aimed at realistic biochemistry, while our approach stresses the regulatory role of morphogenesis within an individual cell. Other modeling techniques incorporating spatial heterogeneity in a cell population in a 2D lattice resulted in the simulation package BacSim [33], the agent-based simulator BSim [34], and the 3D simulator Simbiotics [35]. An interesting study of tumor growth modeling [36] combines cell migration and genetic evolution. These models treat an individual cell as an atomic agent described by a set of (mostly numerical) parameters, A similar but highly specialized approach was applied in [37], but just to simulate the formation of bacterial cell walls. On the other hand, our M systems start the growth from inside the cell and enable understanding of how changes in intra-cellular processes and cell morphogenesis influence the dynamics of the population and overall morphogenesis in an “object-oriented” fashion.

Other models of cell and population growth dynamics approach the subject from an explicit morphogenetic standpoint. A collection of theoretical studies was published in the volume [38], while computational modeling frameworks including 3D ones are presented, e.g., in [39]. Our model of M systems aims at unifying these two approaches, integrating both abstracted biochemical processes and morphogenesis with explicit spatial and geometrical constraints *into a single framework*, with mutual feedbacks between these two crucial mechanisms, as pointed out in [38].

Finally, recent computational methods modeling yet more specialized aspects of intra-cellular processes lead to whole cell modeling, typically combining a large number of different methods into one complex algorithmic framework, requiring tuning hundreds or thousands of individual parameters [10,34,38], with high computational demands and the consequent computational infeasibility. By contrast, here a model is shown where the interplay between biochemical and morphogenetical control of growth processes can result in a much simpler model with just a few parameters, while still providing fairly realistic macroscopic observables consistent with the modeled biological phenomenon, both qualitatively and quantitatively as well.

### 2.3. Implementation of Bacterial Growth Simulation

**Figure 1:**
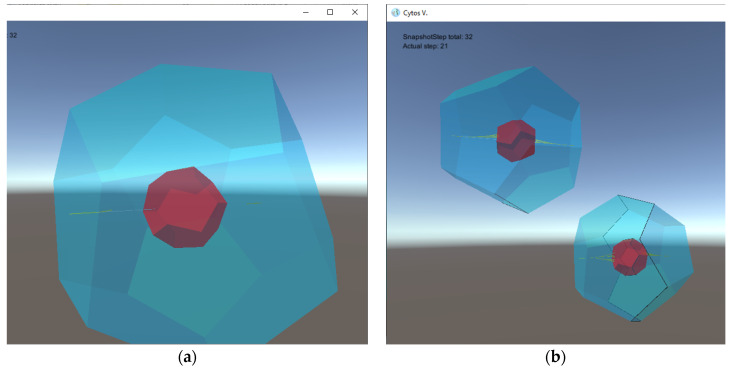
Snapshots of an M system (M_0_) modeling eukaryotic cell membrane (in blue), cytoskeleton and nuclear membrane (in red) formation (**a**), as well as mitosis (**b**) [18,25].

In order to model the formation and growth of prokaryotic cells, a simple M system is described here that uses a septum-like mechanism for cell division. The requirement is that the model should dynamically unfold as follows. First, cell-like walls are gradually self-assembled from input 2D tiles connected with glues. Division starts when the cell reaches a certain “mature” size, not specifically preset in the model, but arising as an emerging property from certain conditions that develop during the self-assembly process. Once the septum is formed, it separates the cell into two subregions, which will develop into the equivalent of daughter cells, as shown in Figure 1. This division re-creates the initial conditions so the cycle can recursively start all over again and develop by the same rules. Actual bacterial cell growth and division consume nutrient resources, here represented by certain objects floating in the environment, such as small molecules or protions. An actual bacterial cell population continues to grow at an exponential rate as long as resources are available in the surrounding environment, as illustrated in [40,41]. The M system simulation is simplified from the biological reality in that there is no primary and secondary septum, but a single (final) septum is formed instead.

**Figure 2:**
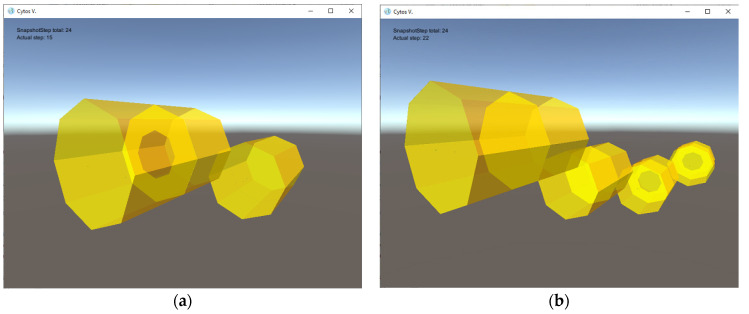
Snapshot of the M system M_bac_ modeling bacterial growth processes with cell division occurring through septum formation (with octagonal tiles). The large octagon in (**a**) has completed its growth in (**b**).

The precise definition of the assembly model M_bac_ consists of the following key components, in accordance with the definition of an M system in the previous section:

- four types of 2D *tiles* that build cell walls and septum components; for the sake of simplicity, these tiles are given polygonal shapes, and hence the resulting cells membranes are shaped as dodecahedra with octagonal sides, as illustrated in Figure 2 (other tiles can be used to produce assemblies with more complex shapes, if desirable);
- three types of small auxiliary *rod-shaped tiles* controlling cell division;
- two types of *floating objects*: the first one (denoted by *a*) contained in the environment with a pre-defined concentration and serving as a nutrient, and the second one (denoted by *s*) serving as a signal molecule controlling the cell division process;
- one type of *protion* located in the tiles of the simulated cells controls the flow of nutrients from the environment into the interior of a simulated cell;
- Nine rules: one *metabolic* rule enables the transport of nutrients into cell interiors; six *creation* rules build tiles and consume nutrients; *one destruction* rule annihilates small auxiliary rods; and one *division* rule concludes the process of cell division when the septum formation is complete.

The full technical description of an M system model is available online at the M systems web page, http://sosik.zam.slu.cz/msystem/Appendix.pdf (accessed on 20 May 2022).

Once these definitions are in place, the model can be easily implemented within an M system simulator, *Cytos* [18]. This software package *Cytos* is now freely available in open source (see the Supplementary Materials section) and can be used to experiment with M systems, such as M_bac_.

In a series of experiments on *Cytos,* an M system was seeded with a single tile in appropriate environmental conditions to analyze the growth of the three selected bacterial species in Table 1. Models for the three species differed in that they were trained by changing the constant for the rate of diffusion of the a- and s-objects to produce results consistent with the experimental data in Table 1. Each simulation was run 100 times, starting with a single cell and with a limited amount of nutrients (a-type floating objects) available. Both doubling times, real and simulated, were observed under limited nutrient amounts, which cause the growth to stop after a certain time without external intervention.

## 3. Results

**Figure 3:**
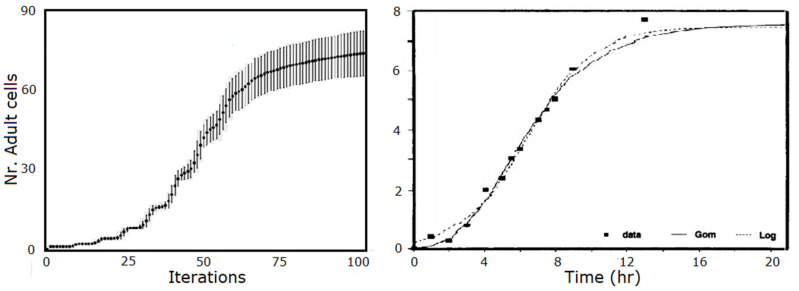
Growth profile of simulated bacterial cells in M_bac_ (**left**). The *x*-axis is the number of iterations simulations (with stds). From the results in Table 2, one iteration corresponds to 1 min in real time. The **right** figure shows the results fitted on *L. Plantarum* data with the Gompertz and logistic models, considered the best prototypes according to [41].

To validate this M system, models for three species of bacteria in Table 1 were built and assessed using the doubling time and growth profile required by the model to that of the corresponding actual bacteria, as shown in Figure 3. This Section shows how certain macro-properties of the resulting cell and membrane assemblies exhibit a number of properties akin to those exhibited by actual living bacterial colonies. Two important properties are illustrated. First, the resulting *dynamic profile* of growth of a population of cells. Second, the *effect of injuries* inflicted on the model as a result of (unexpected) interaction with the environment or agents (in this case, antibiotics) within this environment. 

### 3.1. Bacterial Growth Profiles

Table 2 shows the census of the bacteria grown over time corresponding to the species in Table 1, as well as the corresponding statistics averaging the number of bacteria at the end of a run.

Since experimental observations have demonstrated that the generation time of actual bacteria is on the order of one minute [42], one iteration in the simulation corresponds to 1 min in real time. Since growth is exponential, the number of bacterial cells *B_t_* at time *t* is about

$$B_{t}=B_{0}e^{Kt}$$

where *B*_0_ is number of bacteria at time *t = 0*. The doubling time calculated as *t* (the time which elapsed between *B*_0_ and *B_n_*) divided by the number of generations *n*, is G = *t/n* = *t* log 2/(log *B_n_* − log *B*_0_). In the simulations, *B*_0_ = 1 and *n* = 100, 150, 400 iterations, so the final average population sizes are *B_n_* = 74, 76, 85, respectively, for the species in Table 1, so that the growth rate is *K* = log_2_(*B*_*n*_)/*t*, and the doubling time is *t*/log_2_(*B_n_*), as shown in Table 2. Similar results can be obtained for the other two species in the sample. *Thus, there is a very good agreement between the generation time obtained with the simulation values and those observed for the three actual bacterial species*, as shown in Figure 3, left. Thus, the simulation generation time also agrees with the observed generation time in the order of one minute, i.e., M_bac_ is fairly realistic populationwise, biologically speaking.

From a more principled standpoint, the model produced behavior that is in very good agreement with that of models that have been deemed statistically (using a t- and an F-test) the best prototype models for bacterial growth: the traditional differential equation-based Gompertz models and a logistic model shown in Figure 3, right (as concluded by [41] after a comparative study of a number of them on several species as well).

### 3.2. Robustness Properties of M Systems

As mentioned in the introduction, self-healing and/or resilience of a membrane after certain damage is a characteristic property of living organisms that has not been sufficiently addressed in mathematical or computational models of biological growth. This property can also be defined as the robustness of an organism subjected to environmental or other damage. An important exception is the research in algorithmic tile self-assembly with several studies devoted to this topic, summarized for instance in [24,43].

The conceptual framework of M systems can contribute both theoretical and experimental insights into this topic as well. It is noted that wound healing has a very specific meaning in biology, namely it is the process that repairs a loss or damage to a piece of tissue or organ [44]. Nonetheless, the same term will be used in a more generic sense of repairing any component of a cell, and in particular, will apply it to models of bacterial growth, although bacteria themselves have no distinguishable tissue or organs other than the building tiles. Here we simulate the effect of several types of antibiotics targeting bacterial membranes, and compare the results with *E. coli* population decay under the effect of increasing concentration of antibiotics in [45].

The authors of [18] initiated a rigorous study of robustness in M systems using the concept of an *injury* to the system and demonstrated that the system M_0_ is capable of self-healing of degree 10, as defined below. For example, an injury to the system M_0_ mentioned above could be knocking off one or several tiles that have just attached, or it could be damaging some protions. Other types of injuries included breaking bonds between attached tiles without destroying them, actually deleting a previously added tile (thus creating a hole in a membrane), or sudden appearance of floating objects inside a membrane. The self-assembling nature of the system will simply cause it either to revert to a previous configuration, or to detach a piece of the system altogether. In most cases, the missing part can be re-assembled as it did before, even if the sequence of steps may be different (due to the nondeterministic nature of M_0_), and the new mature individual will bear only small differences compared to the individual before the injury, while keeping the original characteristics of the (uninjured) original M_0_. This property was verified quantitatively in [18], where results of a number of simulations on *Cytos* with injuries to the system M_0_ were described. Interestingly, a certain number of injuries to the cytoskeleton elements led to a small increase of the survival ratio, but the system eventually collapsed when the number of injuries exceeded a critical threshold.

Analogous results were obtained for the M_bac_ system. To better understand these results, a general framework of self-healing in M systems can be defined here to keep this paper self-contained and because it appears to be characteristic of M systems in general. The framework uses the mathematical concept of a directed graph (or just digraph) as a set of vertices (or nodes) together with a set of arcs (directed edges) connecting them and indicating certain kind of relationships between them, as illustrated next. The vertices and edges can represent completely arbitrary objects. Nodes below a vertex represents the overall state of the M system at a given point in time, and the arcs describe possible transitions of the system to other states induced by rules of interaction.

#### 3.2.1. What Is Self-Healing?

**Figure 4:**
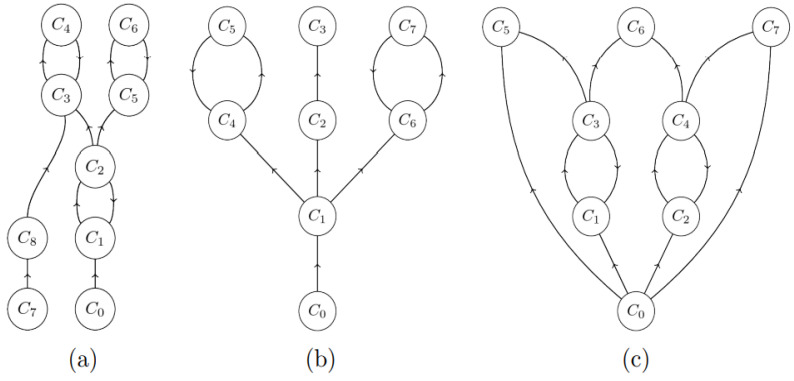
Examples of configuration spaces of M systems with homeostatic components. The root (initial condition) is denoted C_0_. (**a**) The entire graph is a single *h*-component. Nodes C_7_ and C_8_ are unreachable from the root but still belong to the *h*-component. (**b**) A graph with three *h*-components {C_2_-C_3_}, {C_4_-C_5_}, {C_6_-C_7_}. Nodes {C_0_-C_1_} form the morphogenetic phase M^M^. (**c**) The entire graph forms a single *h*-component.

In this section we briefly recall the concept of self-healing introduced in [18] and further elaborated in [46]. A *configuration* (or *state*) of a given M system is a full description or listing of all the objects present in the system at a given time, including their location in the container space (3D space) and their attachments to one another to form substructures (such as membranes or cytoskeletons). The application of a rule changes the current configuration of M, if objects within are close enough to one another to trigger it, or just as a result of Brownian motion. Two configurations are regarded as *equivalent* if they contain exactly the same objects and substructures, and exactly the same rules will apply to either one at the next time step. The *computation graph* of M is a directed graph (digraph) M* whose nodes are *all* the possible distinct configurations (i.e., equivalent configurations are represented by a unique node) obtained in any (randomly chosen) computation of the M system from the initial configuration as the root. The *arcs (x, y)* of M* are all the possible one-step transitions *x => y* of the M system among these configurations as a result of application of the successive interaction rules (*x*/*y* are then called *immediate* predecessors/successors, respectively). Furthermore, M* also includes all other possible configurations (and their interconnecting arcs) having a valid sequence of transitions to some node of M, even if these configurations may not be reachable from the root. The resulting graph is also called the *configuration space* M* of the M system. (Mathematically, M* is obtained as an inverse transitive closure of M in the general digraph consisting of all possible configurations and single transitions by reversing the arrows in the digraph). For example, Figure 4a shows a configuration space where nodes C_7_ and C_8_ are unreachable from the root, but when caused to enter (by damage to the system), lead the M system back to some “normal’’ configuration. 

A *homeostatic component* (or simply *h*-component) is, intuitively, a segment of the configuration space M* (a subdigraph) that, once entered, cannot be exited by following arcs of the successor relation, and which may allow for a cyclic behavior of the M system. The union of all such *h*-components of M* forms the *homeostatic phase* M^H^ of M. Each *h*-component forms a class in the equivalence relation on the set of nodes of M* defined inductively as follows:

- the transitive closure C^ of each directed cycle C belongs to an *h*-component, i.e., it consists of all nodes that are reachable from a node in the cycle following arcs in C using the successor relationship. For example, the closure of C_3_-C_4_ is itself in Figure 4a, while it is the entire graph in Figure 4c.
- each leaf node (one not containing any successors, such as C_5_, C_6_, C_7_ in Figure 4c) in M* belongs to an *h*-component;
- each node *x* whose transitive closure *x^* intersects with a single *h*-component belongs to this *h*-component.

It is clear that when two such components C_1_^ and C_2_^ overlap, then they must both be part of a single *h*-component due to the transitivity of equivalence. To complete the decomposition of M*, the nodes *not* belonging to any *h*-component form an additional equivalence class, called the *morphogenetic phase* M^M^ of M. Figure 4 shows examples of *h*- and *m*-components in simple configuration spaces.

Based on the concept of *h*-components, two biological concepts, adulthood and injury, can now be defined precisely. An *adult* individual in M is one that has reached the homeostatic component M^H^. An *injury* to a morphogenetic system M is a transition of the system given by an ordered pair of configurations (*x*, *y*) such that *y* cannot be obtained from *x* by a *valid* application of any one rule of the system M, i.e., caused by unprescribed events outside the system. The *degree* (or *severity*) of the injury is the undirected graph-theoretic distance between *x* and *y* in M*, i.e., the minimum number of arcs along immediate successors necessary to reach *y* from *x*. It is observed that an injury may not always mean the destruction of a part of the system by an external agent, but it may be also an “irregular’’ attachment or addition to it that could not be added by a single rule application. An injury could even force a transition of the system to a state outside its configuration space. However, the definition implies that no such injury is sustainable, as the system can then never return to the same *h*-component.

An injury (*x*, *y*) is *sustainable* if both *x* and *y* belong to the same *h*-component, i.e., the system would have reached that configuration from a similar development from the injured state anyway. The system is *self-healing* (of degree *m*, respectively) if and only if it can sustain a random injury (of degree at most *m*, respectively) to any homeostatic node with probability of at least 0.5.

These definitions are not only intuitively meaningful, but also can be used to deduce logically necessary properties of self-healing that are inescapable and much more precisely quantifiable, as shown next.

#### 3.2.2. Self-Healing Properties of M_bac_

These general definitions enable inquiries about self-healing properties of an M system. For example, the system M_0_ in [18] is not completely self-healing (of any degree), even if restricting its computation to a single cycle of a process analogous to mitosis. Although the growth of its tiles and cell formation is deterministic, the mitosis process is not, as it is controlled by randomly distributed floating objects, and hence it can produce different results in different runs. Therefore, each mitosis creates two or more *h*-components of a comparable size, plus there are nodes of the morphogenetic phase M^M^ preceding *h*-components in the computation graph. Nevertheless, M_0_ is still self-healing to degree 10.

On the other hand, general properties of self-healing for arbitrary M systems can be established, as illustrated by the following proposition strengthening a result in [18].

**Proposition** **1.** *Every M system with a dominating h-component that occupies at least 71% of all the nodes in its configuration space is self-healing of any degree.*

This statement can be verified as follows. Any randomly chosen homeostatic node belongs to the dominating component with a probability of at least 0.71 (because of the ratio of nodes in the component to all nodes in the graph). Any injury to that node will lead the system to the same component with the same probability. Hence the probability of conjunction of these two independent events, i.e., a random injury of any degree to a homeostatic node resulting in the same *h*-component, is larger than 0.71^2^ > 0.5. Hence the system is self-healing of any degree (actually, the necessary condition could be further elaborated, depending on the number and size of *h*-components of the M system). This argument also illustrates that, as defined, self-healing is a *structural property* of the system, not really depending on a specific configuration, but rather on the global dynamics (the computation graph) driving the overall behavior of the model.

It is clear that most M systems, as it happens with most living systems, are vulnerable to certain critical injuries that they cannot heal, while they may be quite robust to other kinds of injuries. It may be typical that each such system can sustain, with a reasonable probability of survival, certain injuries, as illustrated in [24] for the M system M_0_ simulating division of eukaryotic cells controlled by the growth of a cytoskeleton. Here we extend this study to the case of the self-healing M system M_bac_ described next, which simulates septum-controlled growth of prokaryotic cells.

**Figure 5:**
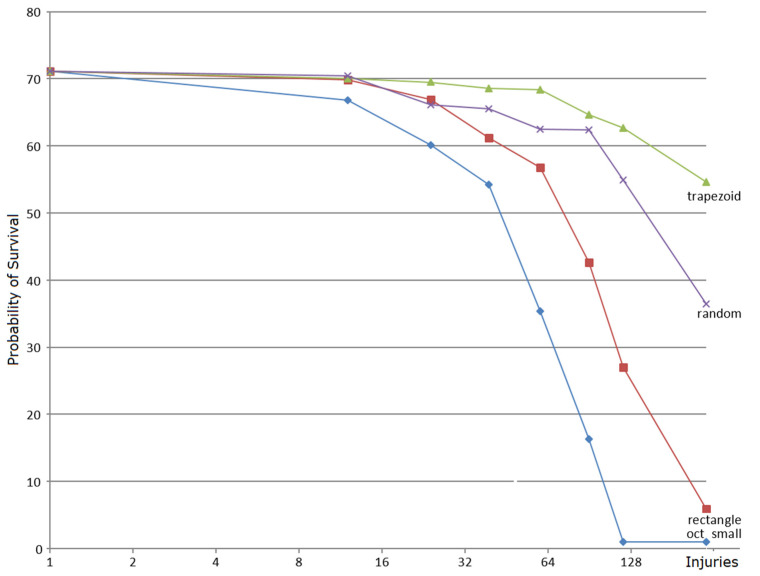
Assessment of robustness of the M_bac_ system. The *x*-axis shows the total number of injuries of four kinds of sensitive components inflicted to the system. The *y*-axis is the probability of survival when *x* injuries of each kind are inflicted on the system.

In a set of experiments, adult individuals generated by M_bac_ were subjected to a random number of injuries, including deletion of one or more tiles of several different types from a cell, deletion of a tile in a membrane, and/or consequent deletion of protions placed on it, which caused intracellular communication to slow down. The injuries were inflicted at random times to randomly chosen parts, prior to division with a septum. Each experiment was run 100 times with the amount of nutrient supplied unlimited in all cases. Figure 5 shows the probability that the cell will sustain those injuries, i.e., still reach a homeostatic stage. The probability drops below 0.50 past 40 or so injuries. To relate these results to laboratory data, it is worth pointing out that the bactericidal mechanism of many antibiotics is based on attacking selected sites on bacterial membranes. This effect is modeled here by “injuries” to membrane tiles. For instance, the results and graphs of an *E. coli* population decay under the effect of increasing concentration of several types of antibiotics (as reported in [45], Figure 1), show remarkable similarity to the results in Figure 5.

**Figure 6:**
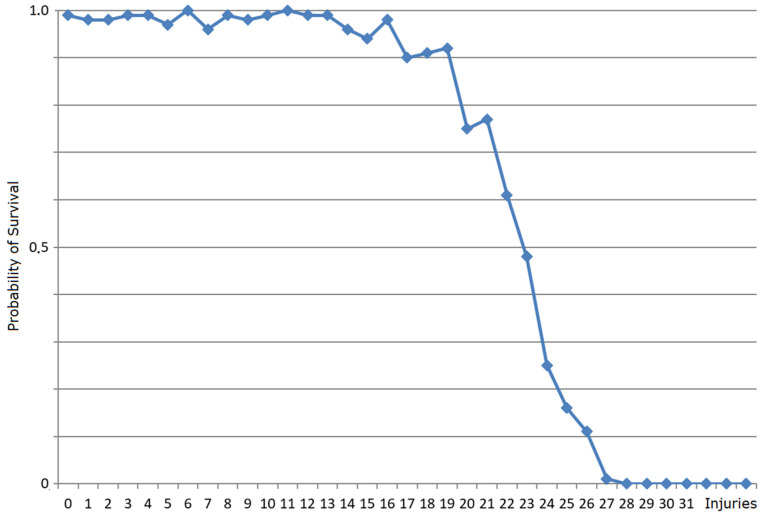
The *x*-axis shows the number of simultaneous random injuries (of any kind) to any of 34 components in an adult individual. The *y*-axis shows the probability of survival to adulthood and reproduction, under abundant nutrient supply, averaged over 100 runs of the experiment. This probability drops under 50% with over 23 injuries.

**Figure 7:**
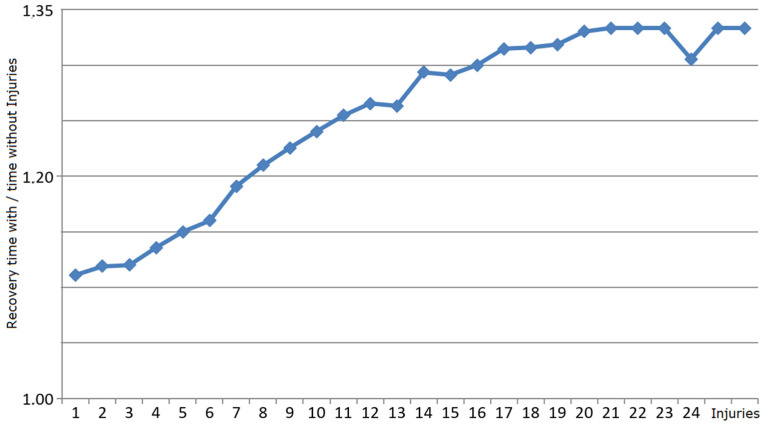
Relative time to recovery of M_bac_ grown individuals. The *x*-axis shows the number of injuries (of any kind) and the *y*-axis shows the ratio of the recovery generation time relative to the normal generation time without injuries in order to reach adulthood. The *y*-values are averages over 100 runs in the experiment.

In a second set of experiments on robustness, an adult individual model of an *E. coli* cell generated by M_bac_ was subjected to a number of simultaneous one-time injuries to randomly chosen parts of the cell, all inflicted at the same time and just before cell division. The experiment was repeated 100 times with the amount of nutrient supplied unlimited in all cases. In the cases when the injuries were sustained, i.e., the cell recovered, the generation time can be compared to the time it would have taken the same individual to continue to grow without injuries. The results are shown in Figure 6. It turns out that at about 23 injuries, the number of successful recoveries out of 100 experiments was already quite small, which resulted in fluctuating values for about *x =* 24 injuries. For values x ≥ 27, the cell never recovered. Likewise, Figure 7 shows the probability of recovery with the number of simultaneous random injuries (of any kind) to any of 34 components in an adult *E. coli* individual cell model.

Finally, similar results were obtained (but not presented here) in experiments performed for variations of the M_bac_ model simulating bacteria with doubling time for the other two species of bacteria shown in Table 1, as described above.

## 4. Discussion

This work presents a family of M system models M_bac_ capable of assembling certain membrane structures from a small number of geometric elements, including tiles, protions, and small objects by interacting through rules prescribed *a priori*. Each of these elements has specific properties stipulating how it interacts with the different elements of the system in close proximity, including itself. These dynamical structures are capable of replicating through the formation of a septum-like wall, despite the fact that none of that was explicitly “programmed” in the interactions. Remarkably, their growth profile closely resembles that observed in different kinds of live bacteria, obtained by adjusting some parameters in the model. Furthermore, the behavior of the resulting population of structures at the macro-level, such as the doubling time of the simulated bacteria, matches very precisely those of three different species of bacteria.

More generally, M systems are a fusion of well-known membrane/P-systems [19] and self-assembly models [23,24]. They are enhanced also by taking into account physically relevant features such as geometric shape and location of their components, hitherto ignored in most mathematical and computational models, but implicit and important for actual biological organisms. M systems make use of objects that abstract certain biological and chemical objects and their properties, such as ions, proteins, and traffic across membranes channels (such as Ca^2+^ channels). These features enable self-assembly of simulated membranes capable of trafficking objects between the environment and the regions of the space on each side of the membrane. The function and dynamics of these objects and membranes are solely based on physically plausible rules of local interaction akin to catalytic reactions and protein trafficking, but more abstract in nature. Therefore, these structures exhibit some degree of physical and biological realism, including nondeterministic operation, while being computationally realistic, i.e., amenable to implementation by simulation in silico within reasonable times.

The M_bac_ model can also exhibit *emergent* properties of bacteria to the point that it allows estimation of the actual behavior of the corresponding living organism’s cells at the macro-level, despite the fact that these properties are *nowhere explicitly specified or enforced in the specification* of the model or its components. These properties include self-controlled growth, division of individual structures, and the generation of a population with a growth kinetics close to that of live bacterial populations. Another property of these structures is that the same model also exhibits self-healing properties when subjected to unexpected interactions with external agents (e.g., akin to antibiotics) in the environment. As such, the models are unique in the sense that a *single* model exhibits all of these properties that can only be obtained one at a time with different models.

Furthermore, M_bac_ consists of only 20 or so rules of interaction among three kinds of constitutive objects such as tiles and protein-like (protion) elements. Also of interest is the fact that the information necessary for the assembly and division of M_bac_ simulated bacterial membranes is not specified by the information content of a specialized element, such as DNA in actual bacteria, but that it emerges dynamically from the properties of the M_bac_ building blocks and of their rules of interaction.

## 5. Conclusions

At a higher level, these results demonstrate that M systems provide a framework to simulate in a more abstract and precise fashion some of the properties of living systems and to explore and quantify their logical relationships, such as the life cycle and their resilience in response to injuries inflicted by their environment. For example, they show that self-assembly and interactions of living organisms with their environments are important for self-healing and that these properties can be defined and quantified more precisely. M_bac_ also revealed that self-healing of the simulated bacteria exhibited an unexpected degree of complexity and unpredictability that deserves further exploration.

Although biology is still essentially a science of observation and experimentation, the historical development of other sciences, such as physics and chemistry, shows that they have benefited from a more theoretical and analytic treatment of their subject matter. The results in this paper show that M systems have the potential to be useful for identifying fundamental and general principles and properties governing the morphogenesis of living organisms from simpler building blocks. Thus, this kind of system might inspire the design of molecules or proteins with properties similar to those described here for the M_bac_ system, and afford a closer examination of their capacity to self-assemble, i.e., the design of some kind of artificial life in silico or in vitro.

## Figures and Tables

**Table 1:** Doubling time of a sample of three species of living bacteria [42].

Bacterium	Medium	Doubling Time (Mins)
*Escherichia coli*	Glucose-salts	17
*Streptococcus lactis*	Milk	26
*Lactobacillus acidophilus*	Milk	66–87

**Table 2:** Experimental average doubling times for bacterial cell growth over 100 runs of M_bac_ with a limited amount of nutrients available. Doubling times have been normalized to *E. coli* times (columns 2 and 4) or estimated from the figures in Table 1 (column 3).

Doubling Times Bacteria	Actually Observed (Mins)	Simulation (Iterations/Time Mins)	Simulation Time (Normalized to *E. coli*)
*Escherichia Coli*	1.00	100/16.10	1.00
*Steptococcus lactis*	1.53	150/24.01	1.49
*Lactobaccilus acidophilus*	3.88–5.12	400/62.58	3.89

## Data Availability

The data presented in this study are available on request from the corresponding author.

## Supplementary Materials

The following supporting information can be downloaded at: https://www.youtube.com/watch?v=mvBLeUHCfW8. Video: M system model of self-reproduction of eukaryotic cells. https://www.youtube.com/watch?v=Mu4nY5yzzhQ. Video: Dynamics of M systems for bacterial growth. https://github.com/mmaverikk/Cytos. Software: The Cytos simulation package for experiments with M systems.
